# Opportunities and Challenges in Tunneling Nanotubes Research: How Far from Clinical Application?

**DOI:** 10.3390/ijms22052306

**Published:** 2021-02-25

**Authors:** Xiaoning Han, Xiang Wang

**Affiliations:** Institute of Biomedical Engineering and Health Sciences, Changzhou University, Changzhou 213164, Jiangsu, China; hxn@cczu.edu.cn

**Keywords:** tunneling nanotubes, intercellular communication, heterogeneity, communication efficiency, drug development, clinical application

## Abstract

Tunneling nanotubes (TNTs) are recognized long membrane nanotubes connecting distance cells. In the last decade, growing evidence has shown that these subcellular structures mediate the specific transfer of cellular materials, pathogens, and electrical signals between cells. As intercellular bridges, they play a unique role in embryonic development, collective cell migration, injured cell recovery, cancer treatment resistance, and pathogen propagation. Although TNTs have been considered as potential drug targets for treatment, there is still a long way to go to translate the research findings into clinical practice. Herein, we emphasize the heterogeneous nature of TNTs by systemically summarizing the current knowledge on their morphology, structure, and biogenesis in different types of cells. Furthermore, we address the communication efficiency and biological outcomes of TNT-dependent transport related to diseases. Finally, we discuss the opportunities and challenges of TNTs as an exciting therapeutic approach by focusing on the development of efficient and safe drugs targeting TNTs.

## 1. Introduction

**Figure 1:**
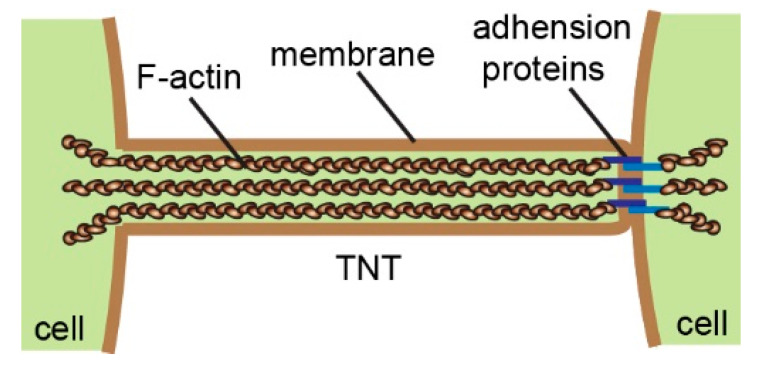
The schematic representation illustrates the structure and general composition of tunneling nanotubes (TNTs).

Multicellular organisms coordinate cell behavior, regulate morphogenesis, and maintain tissue homeostasis by secreting chemical molecules, releasing exosomes, and establishing direct connections such as neuronal synapses and gap junctions [1]. In 2004, the group of Gerdes reported for the first time a new way of long-distance cell–cell communication, tunneling nanotubes (TNTs) [2]. TNTs are tubular membrane structures with diameters of several hundred nanometers, which contain F-actin and, usually, adhesion proteins at one end (Figure 1) [3,4]. Hovering above the substrate, they directly connect adjacent cells up to hundreds of micrometers apart. These unique morphological features make them different from other cellular protrusions, such as filopodia and cytoneme. Numerous studies over the past ten years have shown that TNTs are widely present in various cell types [5,6]. More importantly, TNTs enable the transfer of small molecules, proteins, vesicles, and organelles between cells [2,7,8,9,10,11]. In 2010, we discovered that TNTs mediated depolarization coupling in non-neuronal cells, indicating that TNTs facilitate electrical signal transduction in addition to material transport [12]. Indeed, due to the characteristics of long-distance, high specificity, and multilevel transportation, TNT communication was dubbed the Internet of cells [13].

Subsequent studies have demonstrated the existence of TNT-like structures in tissues, such as mouse corneal stroma, non-neural ectoderms, and zebrafish embryos, suggesting the involvement of TNTs in embryonic development [14,15,16]. A recent work by Alarcon-Martinez and colleagues provided impressive evidence of the presence of TNTs in the retina of living mice and their physiological role in synchronizing the blood flow and neural activity [4]. In pathological conditions, TNTs may provide a new recovery mechanism for injured cells that have a limited ability of proliferation and regeneration [17,18,19]. On the other side, TNTs may transfer survival signals to tumor cells from neighboring cells, which was shown by Osswald et al., that malignant brain tumor cells acquired radiotherapy resistance by establishing a TNT network [20]. Moreover, several pathogens were found to stimulate TNT formation and use them as bridges to propagate between cells [21,22,23]. Thus, it has become a consensus to explore TNTs as therapeutic potential targets [6,24]. However, such an endeavor could take a long way towards future clinical application due to our limited knowledge about these heterogenetic structures. In this review, we will address the opportunities and challenges of TNTs as therapeutics. It begins with an overview of the diversity of structures and biogenesis of TNTs. Then, their versatile functions are introduced, followed by highlighting the communication efficiency of TNTs and the outcomes of TNT-dependent communication. Finally, TNTs as a potential therapeutic target are discussed by focusing on the challenges in the development of TNT-interfering drugs.

## 2. TNTs Are Heterogeneous Structures

### 2.1. Difference in Morphology

TNTs exhibit high variability in their morphology in terms of length and thickness [5,25]. Though the length of TNTs usually changes with the cell spacing caused by cell movement, it ranges from 10 to 100 µm in most types of cells. In a few cases, TNTs have been described as long cytoplasmic extensions up to 300 µm in length [6]. However, these fragile structures may break during prolongation if the pulling force by the cells exceeds the mechanical strength of TNTs [2,26]. In any event, the maximum length of a TNT is crucial since it determines the communication distance between cells. By analyzing the electron microscope images, the diameter of the TNTs was measured from hundreds of nanometers to a microscale [27]. One explanation for such a variation is that TNTs containing microtubules display thicker morphology [19,28,29]. Using cryo-electron microscopy, Sartori-Rupp and his colleagues recently revealed that TNTs were composed of a bunch of ultrathin tubes in mouse catecholaminergic CAD cells and human neuroblastoma SH-SY5Y cells, which are hardly distinguished by conventional confocal microscopy [3]. According to this study, the diameter of a TNT lacking microtubules may be determined by the number of ultrathin tubes. Since the identification of TNTs is still based on their morphological characteristics, the morphological diversity of TNTs has brought confusion to the nomenclature and the literature review on TNT research. The establishment of criteria for the classification of TNTs will be of great significance in this field.

The characterization of TNTs in vitro indicated that TNTs are uniformly F-actin-positive [30], which separates them from other cellular protrusions such as tethers and nanopodia [5]. Surprisingly, F-actin may not be simply arranged in TNTs, as we thought until Anna Sartori-Rupp et al. demonstrated highly organized parallel actin bundles within TNTs [3]. Such a bundle organization could enhance the mechanical stability of these tiny, long structures. As another major type of cytoskeleton, microtubules were also found in TNTs in specific cell types, such as human macrophages, leukemia cells, immature neurons, and apoptotic pheochromocytoma (PC12) cells [19,31,32,33]. Although the molecular mechanism of microtubule-containing TNT (MT-TNTs) formation is still unknown, our study provided evidence that end-binding protein 3 (EB3), a microtubule plus-end tracking protein, moved in MT-TNTs in PC12 cells [19]. Moreover, MT-TNTs were only present in cells at a very early apoptotic stage, suggesting that the formation of MT-TNTs may not be cell-specific [19]. A later study reported that MT-TNTs could establish kinesin/dynein-dependent transfers of mitochondria between cells [18]. Obviously, the difference in the cytoskeleton composition of TNTs not only indicates the existence of different formation mechanisms but also leads to its functional diversity.

### 2.2. Different Mechanisms of TNTs Formation

The importance of F-actin in TNT formation was first proved by our early study showing that a low dose of cytochalasin B, an actin inhibitor, could reduce the number of TNTs in PC12 cells [34]. Even in the MT-TNTs, F-actin plays a dominant role as well, because the treatment of microtubule inhibitors did not significantly disrupt the TNT structures [19,35]. Due to this line of thought, researchers considered that actin regulators and motor proteins were implicated in the formation of TNTs. Many laboratories successively identified several key proteins and signal pathways regulating TNT formation in different types of cells, such as M-sec/ERp29 [36,37,38], p53/Akt/PI3K/mTOR [39,40,41], Myosin10 [42,43], CDC42/IRSp53/VASP [44], and Rab11a/Rab8a [45,46]. Paradoxically, TNTs were also observed in M-sec or p53-deficient cells [42,47]. Such inconsistent results imply that the biogenesis of TNTs may not have a universal molecular regulation mechanism, probably due to their heterogeneity [30].

On the cellular level, two models of TNT formation based on cell–cell interactions in different cell types have long been recognized [48]: (i) the cell dislodgement model, proposing that two adjacent cells retain thin membrane tubes when they move apart [49,50], and (ii) the filopodia interplay model, meaning that TNTs are generated from active cellular protrusions that make direct contact with neighboring cells [30,34,51,52]. Since mechanical forces are required in both models to pull out the tube structure after membrane binding, adequate cell–cell contact is a prerequisite for the generation of TNTs. For example, T cells only formed TNTs after at least four minutes of direct contact [21]. During this “kiss and run” process, cell–cell adhesion proteins provide a sufficient binding force for maintaining the connection at the tips of TNTs [3,53]. In addition, the elongation of TNTs needs to overcome the constraints of the actomyosin cortex on the inner side of the cell membrane, as well as the membrane tension. It should be noted that artificial membrane nanotubes could be pulled out from the cell membrane by magnetic tweezers or atomic force microscopy [54,55]. Thus, we cannot exclude the possibility that some TNTs are generated passively during cell dislodgment. Such passive TNT formation has been observed recently by Staufer et al. showing the formation of membrane tubes during protease-mediated dense cell singularization [56]. In short, the formation of TNTs is regulated not only by actin-relevant molecules but also the mechanical force exerted by cells, which is influenced by the actin skeleton rearrangement, adhesion protein binding, and cell migration.

### 2.3. Where and When Do TNTs Form?

Although the evolutionary significance of TNTs biogenesis is completely unknown, cells under specific physiological circumstances may need these unique structures to establish communication that could not be accomplished by other types of intercellular connections: (i) distant cells connection [4,57,58], (ii) cells migration or invasion [20,59,60], and (iii) heterogeneous cells interaction [17,61,62,63]. Interestingly, the discovery of TNT-like structures between bacteria may provide clues in the study of the putative evolution of TNTs from bacteria to mammals [64]. Whether there are extracellular signals that trigger the formation and directionality of TNTs is still an exciting question. At least, quite a lot of studies have described that the number of TNT-like structures increased in inflammatory and stress conditions, such as pathogen infections [21,31,65], oxidative stress [66], high intracellular calcium concentrations [36], inflammatory signals [67,68], misfolded proteins, and pathogenic amyloid aggregates [8,42,69,70]. Moreover, the tumor microenvironment (hypoxia, acidic pH, hyperglycemia, and serum deprivation), as well as chemo- and radiotherapy-induced reactive oxygen species (ROS) production, leads to more TNTs in tumor cells [39,66,71,72,73,74,75,76]. Additionally, exosomes derived from malignant cells or vesicle recycling induce an increased rate in the formation of TNTs [46,77]. Under these circumstances, cells may respond to the stresses or stimulations by activating signaling pathways that initiate cytoskeleton rearrangement and cell movement, which consequently promote the formation of TNTs.

## 3. Versatile Functions of TNTs

### 3.1. Two Types of Transport Activity via TNTs

TNTs act as intercellular conduits for the exchange of organelles of different sizes, including, but not limited to, mitochondria [4,19], Golgi vesicles [71], and lysosomes [31,78]. These transports are hardly carried out by other types of cell-to-cell interactions and display features of active transportation: (i) requirement of a motor protein in an ATP-dependent manner [17,31], (ii) one-way transportation because of the polarity of motor proteins on F-actin [18,60], and (iii) higher transfer velocity than passive diffusion [22,79]. Moreover, the cargos in TNTs may show controlled movement behaviors, such as stop-and-go and to-and-fro [19,80]. Since our understanding of TNT-dependent active transports remains at a very preliminary stage, many questions need further investigation. For example, how are the cargos recruited towards the openings of TNTs? Is the transport organelle-specific? How do organelles cross the membrane interface between the TNT and connected cell?

TNT-dependent passive transfer mainly occurs to ions, small molecular, and nuclear acid [81]. As a universal second messenger that is easily measured, the intercellular Ca^2+^ flux via TNTs has been widely studied [4,20,54,82,83]. Further work identified that gap junctions were the key proteins which allow the transfer of small molecular or electrical coupling through the border of TNTs [12,20,84,85]. The movement of exogenous materials (e.g., viruses [23,65], bacteria [31], and membrane markers [10,49]) along TNTs could be considered as a special situation of transports. When they bind on the outer surface of TNTs, the transfer was probably accomplished together with the establishment and prolongation of TNTs. On the other hand, virus particles could be carried within vesicles that were driven through TNTs by an active mechanism based on motor proteins [53,86].

### 3.2. Communication Efficiency of TNTs

The amount of data being transfer is determined by the data rate, carrier frequency, and bandwidth [87], which here represent the speed and frequency of transport carried out by a certain number of TNTs. The speed of TNT-dependent communication is mainly dependent on the types of transport. For active transport inside TNTs, the velocity varies from 0.1 to 8 µm/s [11,54,79,88], which is faster than passive diffusion along the membrane tube [89]. An exception is the transduction of electrical signals via TNTs that happen in milliseconds, providing a new concept in the fast communication between distant cells [12,33]. Due to the extremely high length-to-diameter ratio, a TNT is more like a one-lane road for both types of transport. Thus, the frequency of organelles transport driven by the motor proteins is restricted. As for passive transport, in theory, the amount of molecules that diffuse through these submicron tubes is limited. For instance, we did not detect the diffusion of Cascade Blue, a small molecular dye, between TNT-connected normal rat kidney (NRK) cells even after 20 min of dye injection [12]. Moreover, the gap junctions present in TNTs may play a gating role in such communications [6]. Fortunately, a higher bandwidth could be provided by parallel TNTs between a pair of cells that have been shown in our study that recorded a higher amplitude signal through double TNTs [12].

When the communication efficiency of TNTs is examined in the perspective of group cell behavior, it is highly determined by the abundance of TNTs, i.e., the formation frequency and the lifetime of TNTs. The number of TNTs per hundred cells is normally less than 30% in many cell types [10,73,76,90,91]. Most of them are transient structures with an average survival time ranging from a few to tens of minutes [21,34]. Considering the low transportation rate within a single TNT and limited distribution of TNTs among cells, we believe that TNTs are not an efficient route for cell–cell communication. Nevertheless, cells may utilize TNTs for special communication purposes by adopting several strategies. (i) Signaling cascade amplification: a little amount of transferred signaling molecules via TNTs may be amplified by the downstream cascade in receipt cells. Different signaling pathways could be triggered in the receiving cells, such as IP3-induced calcium release [54], depolarization-induced voltage-gated calcium channels opening [12], and Fas signal-induced cell death [92]. (ii) Establishing a network of cells: a group of cells may be connected to each other through TNTs to share signals. Such a phenomenon has been observed in tumor cells that utilize the TNT network to maintain their homogeneous state and enhance their therapy resistance ability [20]. (iii) Multilevel communication: one TNT may facilitate different types of transfers. For instance, organelle exchanges and the diffusion of calcium ions through TNTs may occur in the retinal pigment epithelial cell line and pericytes in the mouse retina [4,82]. In light of the above, the communication efficiency of TNTs should not be neglected when exploring the outcomes of TNT-dependent communication.

### 3.3. Outcomes of TNT-Dependent Communication

Although diverse signals and materials can be transported through TNTs, the subsequent biological events are not fully revealed yet. So far, the most reported is the protective effect on stressed cells, which usually involves the mitochondria, an important organelle associated with apoptosis. Mitochondria were delivered from mesenchymal stem cells (MSCs) to injured cells in acute lung injury, allergic airway inflammation, blood vessels experiencing chemotherapy stress, or dysfunctional pancreatic islet [17,18,93,94]. Injured cells could also be rescued by obtaining mitochondria from neighboring healthy cells via TNTs [19]. Besides, the delivery of functional lysosomes from macrophages to cystinosin-deficient cells displayed a rescue effect by correcting a genetic lysosomal defect [95]. Moreover, brain tumor cells under treatment may utilize a TNT network to propagate intercellular calcium waves and reduce the harmful level of Ca^2+^, thereby obtaining higher resistance to irradiation [20].

Interestingly, proapoptotic proteins transferred via TNTs could induce receipt cell death [92]. Our recent study revealed that prophagocytic membrane markers were delivered from apoptotic cells to healthy cells and induced the phagocytosis of health cells by macrophages [10]. Nevertheless, the currently observed anti- or proapoptotic events induced by TNT-dependent communication are mostly due to the experimental feasibility in detecting cell viability. Undoubtedly, the versatile functions of TNTs result in other cellular consequences as well. It was shown by Connor et al. that cancer cell-endothelial intercellular transport altered the endogenous microRNA (miRNA) profile and phenotype of the recipient endothelial [96]. Therefore, using single-cell profiling, the TNT-dependent transfer-induced alteration in the receiving cells will be revealed at the genomic, transcriptomic, and proteomic levels, which finally provides clues for medical research and applications [11].

Another outcome induced by TNT-dependent communication is the spread of infectious and neurodegenerative substances [23,27,97]. The first evidence provided by the group of Davis showed that membrane nanotubes physically connecting T cells acted as a novel route for HIV-1 transmission [21]. In recent years, viruses of many different families, including retroviruses, herpesviruses, and orthomyxoviruses, have been reported to spread via TNTs [23,98,99]. Besides viruses, mycoplasma, bacteria, and malaria parasites were also found to propagate through TNTs [31,100,101]. Importantly, some pathogens are prone to stimulate the formation of TNTs in infected cells [23]. It raises the question whether pathogens have adaptively evolved a spread model by promoting TNT formation. In a series of studies of degenerative diseases, Zurzolo and her colleagues proposed that prion-like proteins (alpha-synuclein, PolyQ Huntingtin, and fibrillar tau) could effectively propagate between neurons by using TNTs as the predominant mechanism of dissemination [8,22,44,69]. Whereas most of these studies were carried out in vitro, a difficult challenge is how to explore TNT-mediated infections in tissues derived from infected animals and individuals.

In addition to the above cellular events triggered via TNT-mediated transport, the physical connection of TNT itself may participate in cell–cell interactions as well. An interesting study showed that human natural killer cells generated TNTs that contained an immune synapse at the junction point with target cells. The nanotubes could specifically drive the movement of target cells and, finally, lysis them [102]. In another study, airway epithelial cells were demonstrated to directly move along the structure of TNTs, suggesting TNTs may provide spatial information to guide cell migration [59]. By using the microfluidic technique, Marki et al. found that neutrophils rolling on a P-selectin-coated substrate generated nanotubes with one end binding on the substrate. These load-bearing nanotubes aligned along the streamlines of flow stress, implying immune cells may form TNTs as mooring ropes for docking on the endothelial cells in blood vessels [103]. Overall, the current discovery may be seen as only the tip of the iceberg. Further investigation of the roles of TNTs connection together with their interactions with the microenvironment is required for a better understanding of their clinical implications.

## 4. TNTs as Potential Therapeutic Targets

### 4.1. Opportunities Lie Ahead

During the past few years, various studies have suggested TNTs as a potential target for the treatment of tissue injury, tumor drug resistance, and infection (Table 1). As discussed earlier, stressed tumor cells adaptively respond to the tumor microenvironment or therapeutic stress by forming more TNTs [28,72,104,105]. Oncogenes including K-RAS and P53 participate in the activation of proteins regulating the actin cytoskeleton [39,73]. Meanwhile, TNT-based intercellular communication enhances the antiapoptosis ability of tumor cells by delivering mitochondria or dispersing and reducing the cytotoxic factors [106]. As for infections, some viruses can stimulate the formation of TNT to promote spreading between cells [67,107,108]. Therefore, studies have begun to explore the feasibility of disease treatments by blocking TNT formation. The preliminary results have revealed that the inhibition of the TNT-dependent transfer of the mitochondria leads to an increase in chemotherapy-induced cell death or animal survival [74,109]. Additionally, the propagation of HIV viral particles or neurodegenerative alpha-syn fibrils could be impaired when TNT formation is inhibited [67,110]. Thus, it appears that blocking TNT-like connectivity could be a promising strategy for treatment.

Conversely, we need to protect or promote TNT formation in case TNT-based communication allows the delivery of “defensive tools” to repair injured normal cells [128] (Table 1). The ability of cells to exploit TNTs as a bridge to help other cells in pathological conditions might be a natural mechanism for tissue self-repairing. When mesenchymal stem cells were introduced into damaged cultures or tissues, the transfer of the mitochondria from these stem cells was shown to save the recipient cells by recovering their cellular metabolism [17,94], rescuing aerobic respiration [61], or establishing angiogenic capacity [93]. Alternatively, TNT-like structures could remove damaged organelles or autophagosomes to rescue cystinosin-deficient fibroblasts or prematurely senescent endothelial cells [95,129]. In such cases, improving the efficiency of TNT communication will undoubtedly promote the recovery of injured cells.

Another potential clinical application of TNTs is acting as a route for intercellular drug delivery (Table 2). The effective delivery of drugs to target cells is critical for treatment. However, the delivery of biomacromolecular drugs (polypeptides, proteins, antibodies, glycans, nucleic acids, etc.) and drug carriers in tissues mainly relies on slow diffusion and hardly reaches the target cells [130]. Benefiting the ability to transfer macromolecules and vesicles, TNTs could be a novel way for drug delivery within the last few microns in a highly specific manner. Indeed, the transfer of the chemotherapeutic drug doxorubicin via TNTs has been observed in pancreatic, ovarian, and lung cancer cells [29,60,131]. Furthermore, the TNT network may be employed as an efficient way for the redistribution of antitumor drugs among connected tumor cells [131,132]. Nevertheless, extensive investigations are required to elucidate the mechanism of exogenous material transport through TNTs from binding and movement to the final destination in the receipt cells, as well as the overall delivery efficiency. According to the studies showing the quantum dot (QDs) transfer via TNTs [88,133], QDs may be a suitable tool to track drug delivery from donor to recipient cells through TNTs in vitro and even in vivo.

### 4.2. Challenges in TNTs as Therapeutic Targets

Although the clinical benefits of TNT-mediated intercellular communication have reached a consensus in the field of TNT research [6], there is still a long way to go due to the challenges in developing effective and safe TNT-interfering drugs. To date, very few drugs (e.g., arachidonic acid and doxorubicin) have been proven to enhance the formation of TNTs in microvascular endothelial cells or pancreatic cancer cells [29,131,142]. Comparing the efforts that promote TNT formation, the development of TNT inhibitors seems more feasible. Actin inhibitors (e.g., cytochalasin, latrunculin, and tolytoxin) have been widely used in in vitro studies [10,34,82,143]. However, these compounds cannot be applied as medicine due to their cytotoxic effect on the cellular cytoskeleton in tissues [144,145]. Since the safety of TNT-interfering drugs should be a priority consideration, the discovery of low-toxicity drugs will be the first step towards clinical application. To solve this issue, a high-throughput screening system is required to identify drug candidates based on the assessment of both the cell viability and TNT number after the drug treatment. A pioneer work was performed by Hashimoto et al. to search TNT inhibitors using commercially available instruments, which combined a cell culture array on a multi-position scanner, a drug loading system with chemical compounds databases, and imaging software for the quantitative identification of TNTs [37].

Drugs specifically targeting the proteins that regulate TNT formation may belong to those with less cytotoxicity. In a few studies, the number of TNTs could be reduced by the shRNA (short hairpin RNA) -mediated knockdown of CD38 in human multiple myeloma cells [74], impairing the IL-10 (Interleukin-10)/STAT3 (Signal Transducer and Activator of Transcription 3) signaling pathway in human macrophages [67] or blocking beta-CaMKII (Ca^2+^/calmodulin-dependent protein kinases II) in the neuronal cell line [110]. However, it is difficult to find a broad-spectrum inhibitor because of the heterogeneous mechanisms of TNT formation. An alternative strategy is to abolish TNT connections by blocking cell adhesion proteins (e.g., cadherin) [53]. Therefore, it is worth testing the effect of cadherin antagonists such as synthetic linear peptides, synthetic cyclic peptides, and non-peptidyl peptidomimetics [146] on the number of TNTs in various cell types. Moreover, several studies have reported on the formation and function of TNTs that appear to be highly related to the expression of connexin 43 [12,20]. In this regard, conventional gap junction blockers may play a novel role in TNT expressing connexins. Finally, as mentioned earlier, TNTs are very sensitive to mechanical forces [2,26]. Therefore, mechanical shock, such as directional ultrasound or low-frequency oscillation, may be a feasible and safe way to eliminate TNTs in tissues. Nevertheless, we are still facing a challenge on how to evaluate the effectiveness of treatment in removing TNTs in vivo due to the lack of identification markers for TNTs [3,5]. Using laser capture microdissection and microproteomics technology, Gousset et al. recently carried out a mass spectrometry analysis of specific proteins in TNTs isolated from catecholaminergic cells. Their path-breaking study demonstrated that glycolysis and ubiquitin-labeling proteins were enriched on TNTs, which were different from other subtypes of cellular protrusions [147]. In this way, TNT-specific protein markers may be found in the near future. By combining with advanced imaging technology, it is possible to develop a standardized approach to assess the effects of drugs on TNTs in tissues.

## 5. Conclusions

Over the past decade, there has been significant progress in the investigation aiming to understand the biology of TNTs. Numerous studies of different cell systems revealed the heterogeneous nature of TNTs that involve different mechanisms of formation and diverse functions. Although the outcomes of such heterogeneity are still not fully clear, the roles of TNTs in tissue repair, cancer, and infectious and neurodegenerative diseases indicate a wide range of implications of TNTs in the field of biomedical research. Thus, how to use TNTs as a therapeutic target in clinical applications has gradually become the main focus of this field. To close the gap between our current knowledge and clinical applications, we need a deeper understanding of TNT-dependent communication by elucidating (i) specific molecular events occurring in TNTs, (ii) global cellular responses to TNT-mediated transportation, (iii) dynamic interactions of TNT-connected cells with their microenvironment beyond chemical signals, and (iv) the communication efficiency of TNTs from a macroscopic perspective. Moreover, we are still facing challenges in developing efficient and safe drugs targeting TNTs. Nonetheless, the recent advances in using advanced technology to identify biomarkers of TNTs and screen TNT inhibitors may open up a new era for TNT research and translational medicine.

## Figures and Tables

**Table 1:** Tunneling nanotubes (TNTs) as potential targets in disease treatment.

Treatment	Role of TNTs		References
Inhibit TNT formation	Cancers	TNTs drive tumor cell invasion, proliferation, and intercellular connection to protect cell death from radiotherapy in astrocytic tumor.	[20]
		TNTs in acute lymphoblastic leukemia (ALL) cells and mesenchymal stromal cells (MSCs) promote leukemogenic processes, the proliferation of ALLs, and increase chemotherapeutic resistance.	[111]
		Cancer cell transfer microRNAs via TNTs to endothelium to promote metastasis.	[96]
		Mitochondrial transfer through TNTs from neighboring nonmalignant bone marrow stromal cells (BMSCs) to multiple myeloma cells increase their oxidative phosphorylation.	[74]
		Mutant KRAS promote TNT formation in colorectal cancer cells, inducing intratumoral heterogeneity and invasiveness.	[73]
		Cell fusions via TNTs lead to glioma tumor heterogeneity and promote tumor cell survival against treatment.	[68]
		Therapy against chronic myeloid leukemia (CML) increase TNT formation in bone marrow-derived malignancies.	[91]
		Irradiation in glioblastoma cells promotes TNT formation and cell survival.	[112]
		Astrocytes establish TNT connections with glioblastoma (GBM) cells, thus promote tumor growth and migration.	[113]
		Bladder cancer cells form TNTs to connect others, transferring miR-155 and acquiring a higher proliferative rate.	[40]
		TNTs between macrophages and tumor cells promote tumor invasion.	[114]
		Vemurafenib against colorectal cancer (CRC) enhances TNT formation in CRC cells with increasing therapy resistance.	[115]
		Mitochondria transferred from stromal cell cancer-associated fibroblasts to prostate cancer cells enhance their migration and metastatic ability.	[116]
		Stress promotes TNT formation between prostate cancer cells to resistant treatment.	[76]
	Neurodegenerative diseases	Prion trafficking through TNTs in neurons.	[22]
		Transfer of polyglutamine aggregates in neuronal cells.	[7]
		Misfolded α-synuclein transfer through TNTs inside lysosomal vesicles in neuronal cells.	[69,110]
		Tau transferred inside TNTs connecting neuronal cells.	[117,118]
		Transfer of Huntington disease protein, mHTT, in neurons.	[41]
	Infections	Intercellular transmission of malaria parasites in the mosquito midgut.	[100]
		Transfer of bacillus between Human Macrophages.	[31]
		Transfer of tuberculosis bacillus (TB) and HIV-1 virus in human M (IL-10) macrophages.	[67]
		Transmission of HIV-1 within T cells.	[21]
		Nef HIV-1 increases TNTs and transfer via TNTs from macrophages to T cells.	[43]
		Transfer of influenza virus between lung epithelial cells.	[119]
		Transfer of bovine herpesvirus 1 between bovine primary fibroblasts and oropharynx cells.	[99]
		Transfer of Mycoplasma hyorhinis between NIH3T3 cells.	[101]
		Macrophages initiate fusion via TNT-associated cell connection, resulting in multinucleated giant cells in chronic inflammatory disease.	[120]
Promote TNT formation		Bone marrow-derived stromal cells (BMSCs) protect alveolar epithelia in mice through TNTs against acute lung injury.	[17,18]
		MSCs protect endothelial cells from apoptosis via TNT-mediate mitochondrial transfer.	[121]
		Neural stem cells rescue brain function by formatting TNTs with brain microvascular endothelial cells.	[122]
		Renal CD133+ scattered tubular cells (STCs) protect injured tubular cells (TECs) in rat kidney.	[123]
		TNTs among MSCs help to maintain their stemness and differentiation potential.	[124]
		MSCs improved non-alcoholic steatohepatitis (NASH) lipid metabolism and tissue homeostasis via TNTs in mouse livers.	[125]
		Interpericyte TNTs coupled changes in microvasculature to neuronal activity through the bi-directional transfer of Ca^2+^ in mouse retina.	[4]
		Macrophages deliver lysosomes to cystinosin-deficient cells, leading to tissue preservation.	[126]
		MSCs transfer healthy mitochondria to damaged neural stem cells via TNTs.	[127]

**Table 2:** Transfer of nanoparticles and drugs via TNTs.

Cargoes	Cells	References
Nanocrystals	Human hepatocellular carcinoma cells, HepG2 cells	[134]
Quantum dots (QDs)	Rat cardiac myoblast cells, H9c2 cells	[88]
Fluorescence carbon dots	4T1 cells	[135]
QDs	Human proximal tubular epithelial cells (RPTEC)	[136]
Carboxyl-QDs	Perivascular macrophages	[133]
Silicon microparticles	Human microvascular (HMVEC) and umbilical vein (HUVEC) endothelial cells	[137]
Polymer-based nanoparticle	Hela cells	[138]
Nanotags	Hela cells	[139]
FITC-SiO2 nanoparticles	Hela cells	[140]
liposomes carrying mApoE and chlorotoxin	Glioblastoma (GBM) cell line U87-MG cells and normal human astrocytes (NHA)	[141]
Doxorubicin	Human pancreatic cancer cells: MIA PaCa-2, S2013, CAPAN-1, and CAPAN -2	[131]
Doxorubicin	Macrophage cells RAW264.7 and human non-small cell lung cancer A549 cells	[29]
Doxorubicin	From macrophages to ovarian carcinoma cells	[60]
